# Biomechanical comparison of acetabular fracture fixation with stand-alone THA or in combination with plating

**DOI:** 10.1007/s00068-021-01872-0

**Published:** 2022-01-17

**Authors:** Lisa Wenzel, Sabrina Sandriesser, Claudio Glowalla, Boyko Gueorguiev, Mario Perl, Fabian M. Stuby, Peter Augat, Sven Hungerer

**Affiliations:** 1 Department of Trauma and Orthopedic Surgery, Berufsgenossenschaftliche Unfallklinik Murnau, Prof. Küntscher Str. 8, 82418 Murnau, Germany; 2 AO Research Institute Davos, Clavadelerstr. 8, 7270 Davos, Switzerland; 3 Institute for Biomechanics, Berufsgenossenschaftliche Unfallklinik Murnau, Prof. Küntscher Str. 8, 82418 Murnau, Germany; 4 Institute for Biomechanics, Paracelsus Medical University, Strubergasse. 21, 5020 Salzburg, Austria; 5 Department of Trauma and Orthopedic Surgery, University Hospital Erlangen, Friedrich-Alexander University Erlangen-Nürnberg, Maximiliansplatz. 2, 91012 Erlangen, Germany

**Keywords:** Acute total hip arthroplasty, Anterior column, Acetabular fracture, Revision cup, Suprapectineal plate, Biomechanics

## Abstract

**Purpose:**

A common surgical treatment in anterior column acetabular fractures with preexisting osteoarthritis is THA, which is commonly combined with plate osteosynthesis. Implantation of a solitary revision cup cranially fixed to the os ilium is less common. The purpose of this study was to compare the stabilization of anterior column acetabular fractures fixed with a cranial socket revision cup with flange and iliac peg or with a suprapectineal plate osteosynthesis combined with an additional revision cup.

**Methods:**

In 20 human hemipelves, an anterior column fracture was stabilized by either a cranial socket revision cup with integrated flange (CF = Cup with Flange) or by a suprapectineal plate combined with a revision cup (CP = Cup and Plate). Each specimen was loaded under a stepwise increasing dynamic load protocol. Initial construct stiffness, interfragmentary movements along the fracture line, as well as femoral head movement in relation to the acetabulum were analyzed.

**Results:**

Both groups showed comparable initial construct stiffness (CP: 3180 ± 1162 N/mm and CF: 3754 ± 668 N/mm; *p* = 0.158). At an applied load of 1400 N, interfragmentary movements at the acetabular (*p* = 0.139) and the supraacetabular region (*p* = 0.051) revealed comparable displacement for both groups and remained below 1 mm. Femoral head movement in relation to the acetabulum also remained below 1 mm for both test groups (*p* = 0.260).

**Conclusion:**

From a biomechanical point of view, both surgical approaches showed comparable fracture reduction in terms of initial construct stiffness and interfragmentary movement. The potential benefit of the less-invasive cranial socket revision cup has to be further investigated in clinical studies.

## Introduction

Age distribution of patients suffering acetabular fractures has two peaks. The first one is found in patients younger than 40 years mainly caused by high energy trauma and a second peak in elderly due to low energy trauma, e.g., stumbling falls [1]. For the latter, a systematic review of Daurka et al. showed an increasing number of acetabular fractures due to aging of the population accompanied by reduced bone mineral density leading to osteoporotic bones and therefore to a higher susceptibility for fractures [2]. In addition, preexisting osteoarthritis of the hip is often observed in older patients. Recent literature specified the most common fracture patterns with both column fractures (19%), followed by anterior column and posterior hemi-transverse fractures (17%) and anterior column fractures (17%) according to the Letournel classification [3, 4].

An increasing involvement of the anterior wall has been observed in patients older than 60 years [4]. Therapeutic options for the management of acetabular fractures include conservative treatment, joint reconstruction with internal fixation and total hip arthroplasty (THA) [3]. Open reduction and anatomical joint reconstruction are mainly reserved for patients without signs and symptoms of osteoarthritis of the hip. Conservative treatment is indicated for compliant patients with minor fracture dislocation or a high ASA-classification grade. The option of THA is mostly considered in patients with additional fractures of the femoral neck, pathologic fractures or an advanced osteoarthritis of the hip [5]. A clinical study by Carta et al. investigated THA compared to open reduction and internal fixation in acetabular fractures in elderly patients and found advantages for THA regarding surgical time, length of stay, and quality of life and hip function [6]. In case of fracture stabilization by means of arthroplasty, two different therapy regimens exist. The more common treatment principle involves open reduction and osteosynthesis using plating and immediate or early implantation of a hip arthroplasty to treat osteoarthritis. An alternative, so far less common treatment principle, is the acute total hip arthroplasty, e.g., with a revision cup fixed cranially to the os ilium (cranial socket cup). The advantage lies in a reduction of the surgical trauma and in the single operation, especially in view of the geriatric and multimorbid patient population. Recent studies report on the surgical management of acetabular fractures [7–9], but which treatment to prefer has not yet been adequately investigated in clinical or biomechanical studies. There are hints, that acute primary arthroplasty has positive effects on pain and improved function compared to open reduction and internal fixation [10].

The purpose of this study was to biomechanically compare the stabilization of anterior column acetabular fractures fixed with either a cementless cranial socket revision cup with flange and iliac peg or with a suprapectineal plate osteosynthesis combined with an additional cementless revision cup.

## Materials and methods

Ten fresh-frozen human pelves from three female and seven male donors with a mean age of 75 ± 11 years were obtained from an accredited donation program (Science Care Inc., Pheonix, AZ, USA). All specimens were scanned by quantitative computed tomography and analyzed by a 3D software (Amira, Thermo Fischer Scientific, USA) to assess bone mineral density (BMD) in the acetabular domes. Each pelvis was parted and randomly assigned to both test groups with an equal number of right and left specimens per group.

**Fig. 1:**
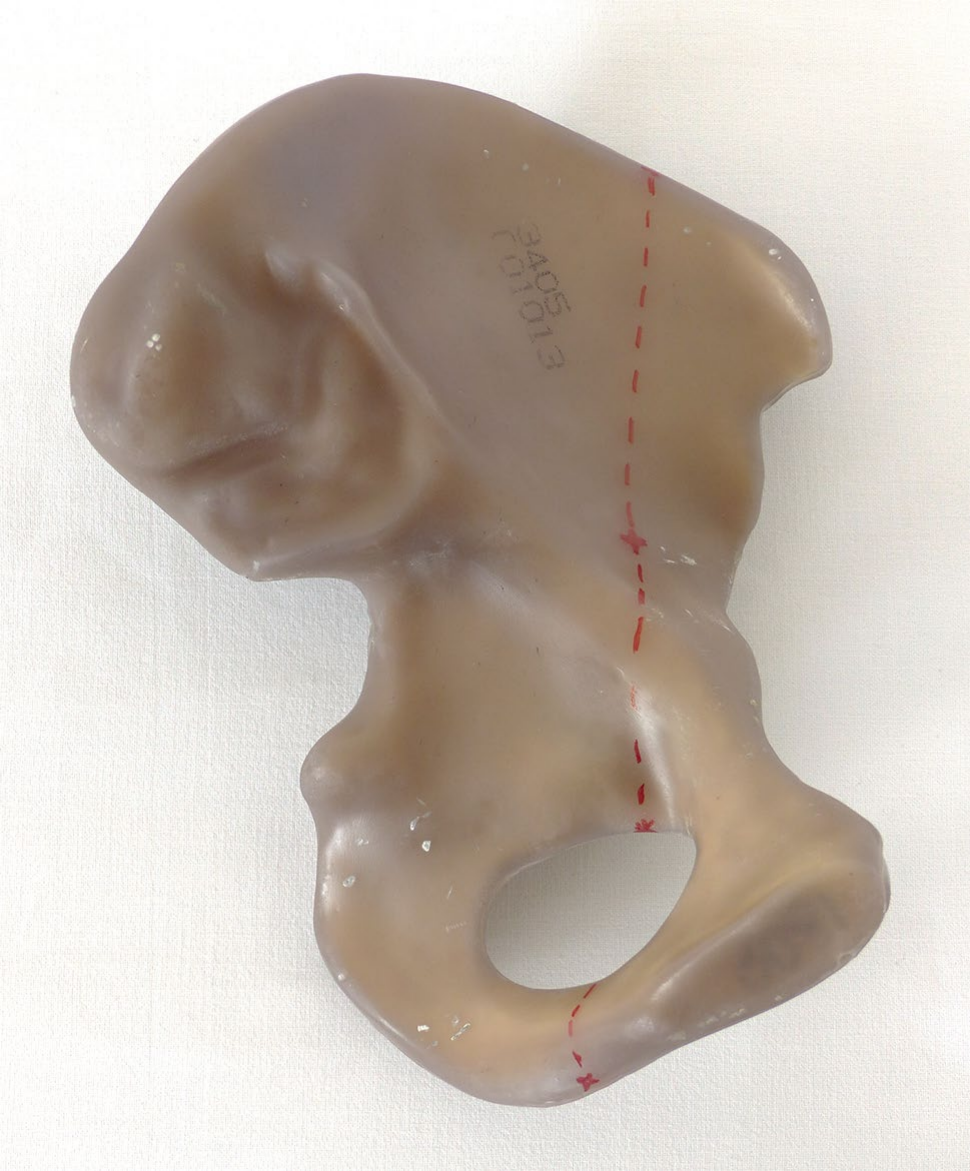
Medial view of a left hemipelvis with the dotted line defining the fracture line of the anterior column fracture

All specimens were kept frozen at −20 °C and thawed overnight prior to preparation. After removing all soft tissue, an idealized fracture line of the anterior column was marked in a strictly reproducible manner along with the following anatomical landmarks: 25% of the distance from the anterior superior iliac spine (ASIS) to the posterior inferior iliac spine (PIIS); 35% between the anterior inferior iliac spine (AIIS) and the deepest point of the greater sciatic notch; 25% of the distance of the acetabular notch; 50% between the superior-posterior edge of the pubic symphysis and the largest bulge of the sciatic tuberosity. The fracture line was continued in direction of the main osteotomy line (Fig. 1).

Before fracturing, each hemipelvis was embedded at the sacro-iliac joint with the axes being aligned according to Morosato et al. to represent an upright standing posture [11]. To provide additional support on the limited embedding zone, five small screws (3.5×20 mm) were inserted into the articular surface of each specimen and the screw heads were covered with embedding material (RenCast FC 53 A/B + Füller DT 082, Huntsman, The Woodlands, TX, USA). After curing of the casting resin into rigid polyurethane, the acetabula were reamed to the defined cup size based on CT scans. In some cases, size adjustment was necessary during the implantation and reaming process. The anterior column fracture was osteotomized according to the marked fracture line with an oscillating saw of 1 mm blade thickness.

### Surgical technique

All implantations were conducted by two experienced surgeons (SH, CG) following the surgical guidelines of the implant manufacturer to limit the inter-observer variability. To guarantee a reproducible implantation process, the embedded part of the specimens was clamped in a bench vice. As a first fracture reduction, the anterior column fragment was temporarily stabilized by a k-wire placed supraacetabular from anterior to posterior. A second k-wire was placed through the spina iliaca anterior superior into the os ilium.

**Fig. 2:**
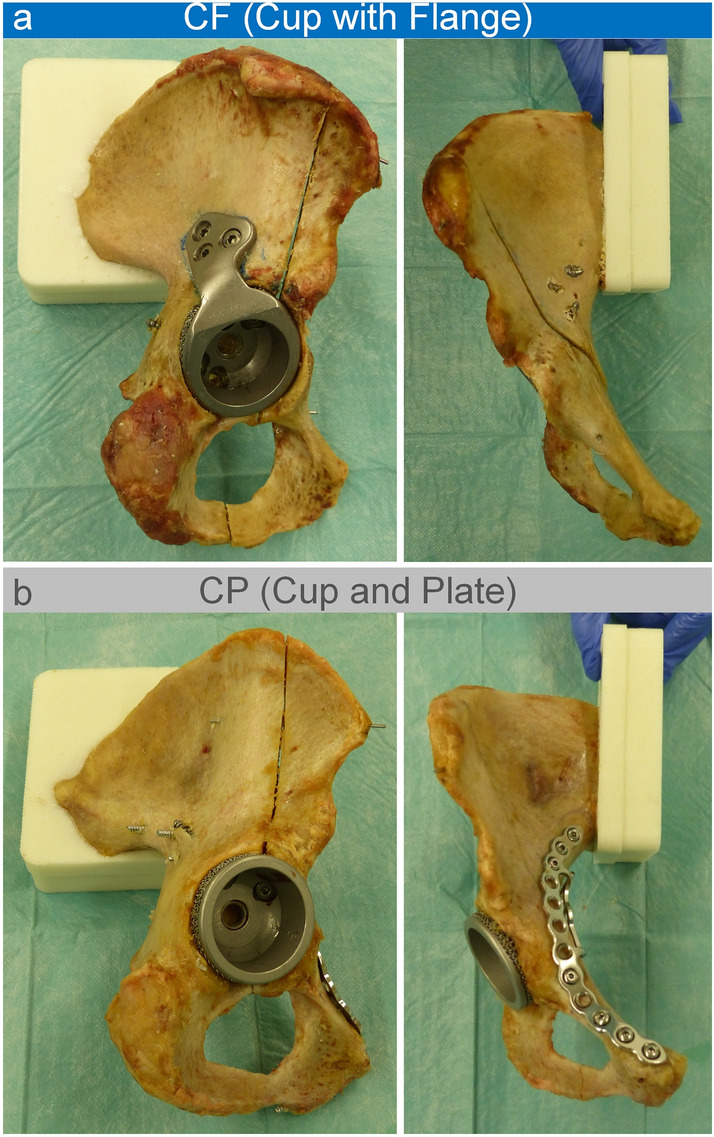
Lateral view of the hemipelvis (left) and view from anterior (right) of both test groups: **a** cranial socket revision cup with integrated flange and iliac peg; **b** revision cup with an additional suprapectineal plate fixation

In the CF (Cup with Flange) group, the fracture was addressed by a cementless cranial socket revision acetabular cup with integrated flange (AQ Revisio M, AQ Implants GmbH, Grevesmühlen, Germany) (Fig. 2a). The flange was fixed by all three screw placement options. The cup was stabilized by two of the possible screw fixation options and it was additionally stabilized by a fixed angle iliac peg into the iliac bone. In the CP (Cup and Plate) group, a quadrilateral surface plate (QLS suprapectineal plate, Stryker GmbH, Selzach, Switzerland) was placed prior to cup implantation. The plate was fixed using three screws in the most anterior and three screws in the most posterior holes. To provide enhanced plate stability, an infraacetabular screw was placed through the thin inferior part of the acetabular corridor into the tuber ischiadicum. In addition to the plate, a cementless press-fit revision acetabular cup (AQ Revisio S, AQ Implants GmbH, Grevesmühlen, Germany) using three screws for fixation was implanted (Fig. 2b). To verify correct implant positions, all specimens were postoperatively CT-scanned and once again after mechanical testing.

### Test setup

**Fig. 3:**
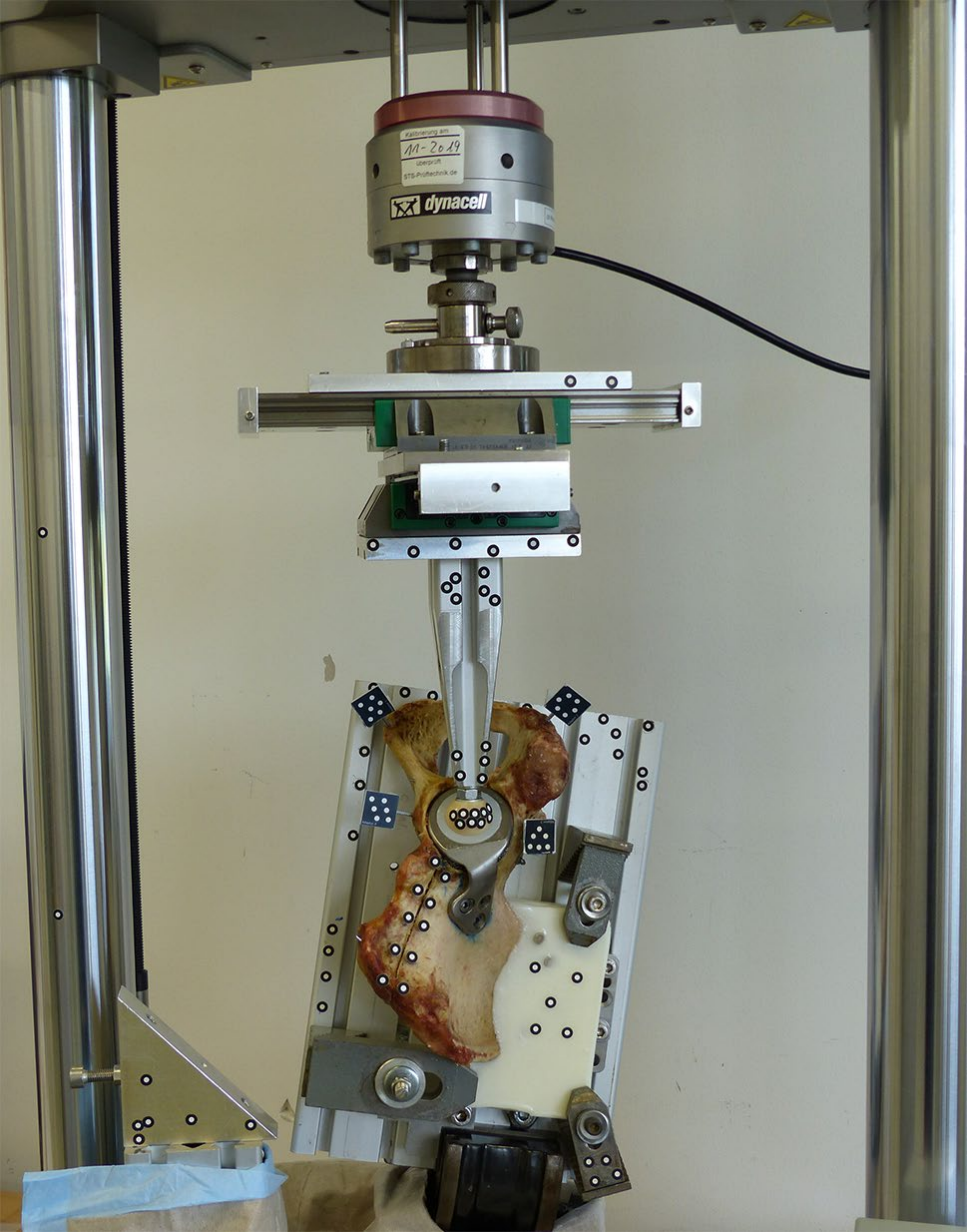
Test setup with the hemipelvis being inverted and tilted to mimic heel strike. The load was applied with an artificial femoral head via a linear slide to reduce constraint forces

The specimens were mounted on an electrodynamic test machine (E3000, Instron, High Wycombe, UK) in an inverted position for better handling (Fig. 3). Load was applied by an artificial femoral head (Ø 28 or 32 mm) that was fixed to the machine actuator and load cell via a linear slide to avoid constraint forces. The hemipelvis was mounted on an aluminum plate in a way that the specimen rested on the embedded sacrum and the symphyseal region. To simulate forces acting on the acetabulum during heel strike, the clamped aluminum plate was tilted in a vice in two planes. Based on gait parameters by Perry, the pelvis is tilted in sagittal plane for 20° hip flexion and in frontal plane for 10° adduction during heel strike [12]. Combining this information with the resulting force vector in the hip joint during heel strike by Bergmann et al. (11° sagittal, 17° frontal), the hemipelvis was tilted for 9° (20–11°) of hip flexion and 7° (10–17°) of hip abduction [13].

Prior to dynamic loading, initial construct stiffness was determined by three quasi-static ramps from 10 to 200 N at a velocity of 0.01 mm/s. After that, the specimens were loaded in a stepwise increasing sinusoidal load protocol starting at partial weight-bearing loads of 50–200 N at a frequency of 2 Hz. The load valley of 50 N was kept constant, while the peak load increased for 50 N after every 1000 load cycles. Termination of the test was defined as actuator displacement of 10 mm, or a maximum load of 3000 N was reached. Interfragmentary movements were measured at the load valley (50 N) and the respective loaded state after each 1000 cycles by an optical 3D motion tracking system (ARAMIS Professional 5M, GOM GmbH, Braunschweig, Germany). Therefore, marker flags were positioned to detect the movement in the acetabular region and small marker points were attached along the fracture gap to detect supraacetabular movement. To investigate elastic and plastic deformation of the femur-acetabulum construct, marker points on the artificial femur head were tracked in relation to the embedded part of the os ilium fragment. Translations were calculated based on a coordinate system that was aligned according to the load axis, simulating the force vector in the hip joint.

### Data analysis

To verify the test setup and load protocol for both implants, one specimen pair was used for pretests, which allows a total number of *n* = 18 specimens (*n* = 9 per group, respectively) for inclusion in the main tests and analysis. Construct stiffness was calculated by averaging the linear portion of the three force–displacement curves. To allow for inter-specimen comparability, interfragmentary movements were analyzed at the highest load step all specimens withstood. Analysis of movements was conducted within the software of the tracking system (GOM Correlate Professional, GOM GmbH, Braunschweig, Germany). Data were tested for normal distribution using Shapiro–Wilk test. Bone mineral density and stiffness data were compared using paired *t* tests and translational movements were compared using Wilcoxon tests. Despite the use of a signed-rank test, the values in the Results section are given as mean ± standard deviation. Correlation of BMD with the amount of interfragmentary movement was analyzed by Spearman tests (SPSS Statistics, Version 26, IBM, US). Level of significance was set to 0.05.

## Results

Bone mineral density was evenly distributed in both test groups (CF: 130 ± 47 mg/ccm, CP: 136 ± 49 mg/ccm; *p* = 0.296). Both test groups showed comparable initial stiffness (CF: 3754 ± 668 N/mm, CP: 3180 ± 1162 N/mm; *p* = 0.158).

**Fig. 4:**
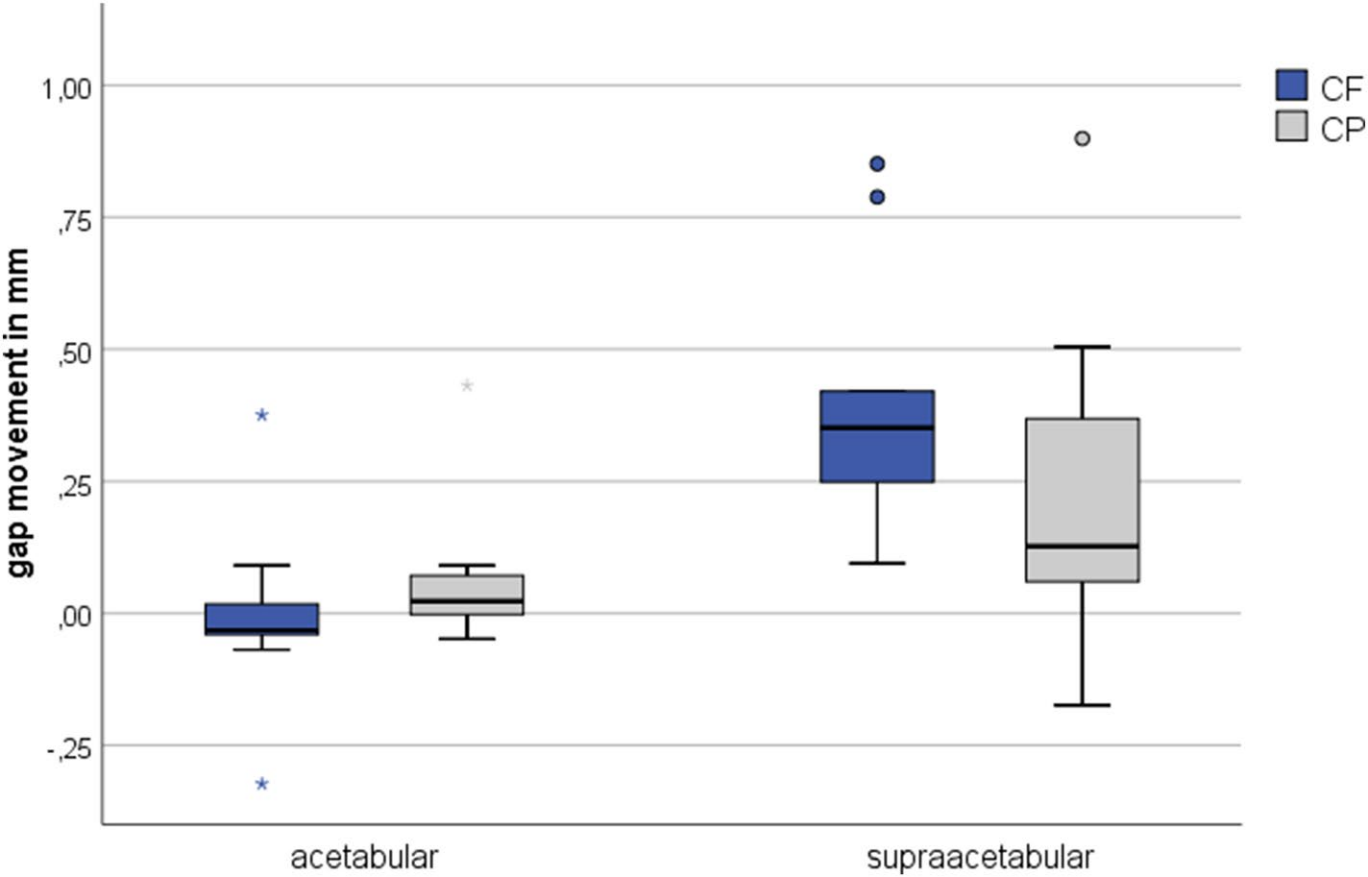
Interfragmentary movement at the acetabular and the supraacetabular region at 1400 N for the CF group (Cup with Flange) and the CP group (Cup and Plate). Negative movement represents closing of the fracture gap. The boxplot shows the median and the interquartile range (IQA) with its outliers and whiskers at 1.5*IQA (o) and 3*IQA (*)

1400 N was the defined load step for interfragmentary movement analysis, as all specimens survived this load level. In the acetabular region, gap movement remained below 0.5 mm and showed no difference between the tested implants (0.00 ± 0.17 mm for CF and 0.07 ± 0.13 mm for CP; *p* = 0.139) (Fig. 4). Negative movement represented closing of the fracture gap, while positive movement symbolized widening of the fracture gap.

**Fig. 5:**
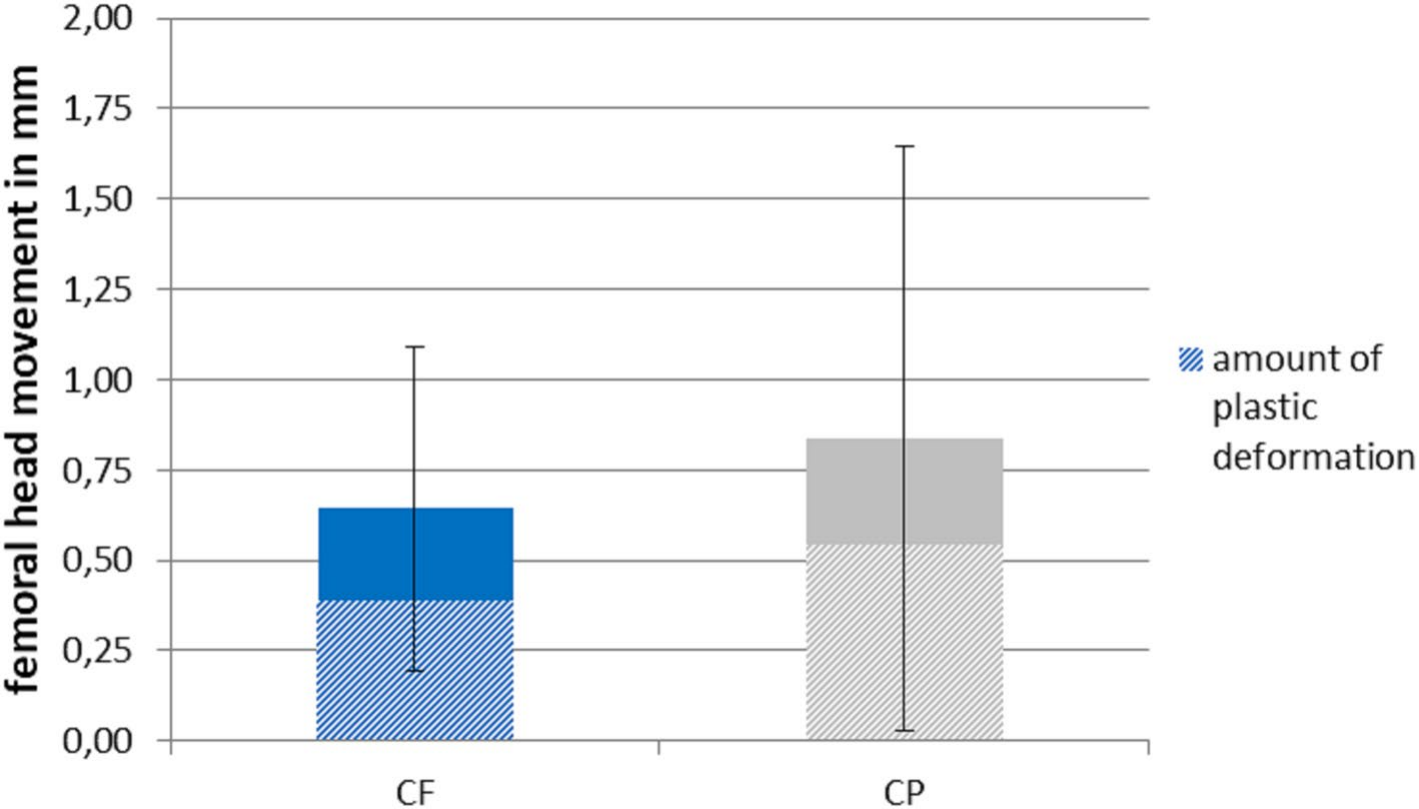
Movement of the femoral head in relation to the acetabulum at 1400 N for the CF group (Cup with Flange) and the CP group (Cup and Plate). The dashed section represents the amount of plastic deformation that remains after unloading the construct to the load valley of 50 N

Movement in the supraacetabular region was approximately twice as high, but did not exceed 1 mm (0.39 ± 0.25 mm for CF and 0.23 ± 0.30 mm for CP). A trend toward increased stability was found in the CP group, which showed higher reduction in movement compared to CF, but these differences were not significant (*p* = 0.051). Movement of the femoral head in relation to the acetabulum was in the range of 0.64 ± 0.45 mm for CF and 0.84 ± 0.81 mm for CP (*p* = 0.260), with a quite high amount of plastic deformation (0.39 mm vs. 0.54 mm, respectively) (Fig. 5).

**Fig. 6:**
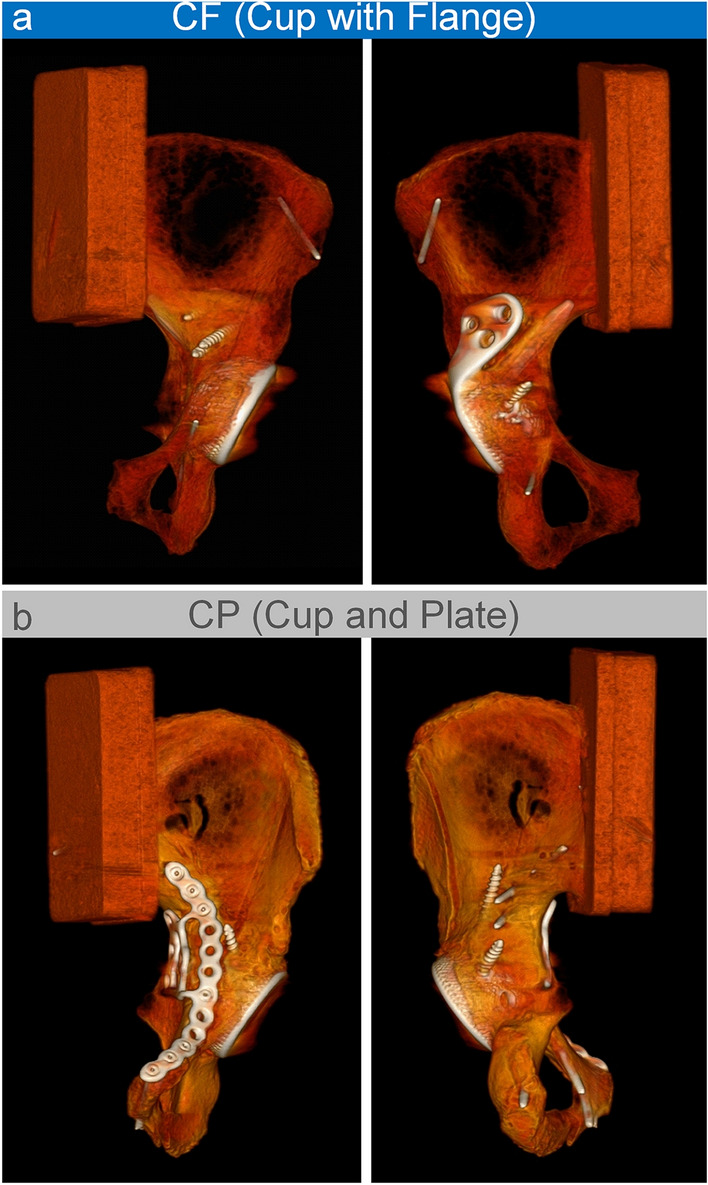
Exemplary post-operative CT reconstructions in medial-anterior view (left) and from posterior (right) of both tested groups: **a** cranial socket revision cup with integrated flange and iliac peg; **b** revision cup with an additional suprapectineal plate fixation

A correlation of BMD with interfragmentary movement was neither detected in the acetabular (*r* = −0.412, *p* = 0.089) nor the supraacetabular region (*r* = −0.413, *p* = 0.088). Seven out of 18 specimens withstood the load protocol up to 3000 N (*n* = 4 CF, *n* = 3 CP). Four specimens failed due to loosening of the cup (*n* = 1 CF, *n* = 3 CP) and the remaining seven constructs failed due to breakage of the bone at its weakest point close to the embedding material. Post-operative CT scans revealed reproducible and exact screw and cup positioning in all specimens (Fig. 6). After mechanical testing, only one cup screw breakage in a CP sample was detected in the position closest to the fracture gap.

## Discussion

In this biomechanical study on anterior column fractures of the acetabulum, the stabilizing effect of two different press-fit revision cups was investigated. Cementless arthroplasty with or without additional plating represents a common surgical management of primary fracture treatment in patients with osteoarthritis of the hip. The novelty of our study is the biomechanical investigation of a cranial socket revision cup with flange and fixation peg compared to a revision cup with additional plate osteosynthesis. Both groups showed comparable results in terms of interfragmentary movements at the acetabular and the supraacetabular region as well as similar amounts of femoral head movement in relation to the acetabulum with mean displacement values below 1 mm. An advantage of the cranial fixed revision cup is the less-invasive surgical procedure as there is only one access needed to implant the cup, but more exposure of the os ilium is needed for positioning and screw fixation of the flange. Additional plating demands a more extended surgical approach with extensive soft-tissue trauma, which also influences the functional outcome depending on the selected approach [14]. However, nowadays, less-invasive approaches like the pararectus approach for anterior stabilization of the acetabulum are available [14]. In case of a two-step procedure, a second anesthesia is necessary, which should be avoided especially in elderly patients increasing the risk of dementia [15]. After total hip arthroplasty, immediate full weight-bearing should be possible, especially for elderly patients without the ability to comply with partial weight-bearing restrictions. Moreover, full weight-bearing has the potential to lower the risk, e.g., for deep vein thrombosis and prevents muscle loss due to immobilization [16]. Weaver et al. reported that primary total hip arthroplasty leads to an improved function and reduced pain compared to open reduction and internal fixation [10]. However, plating is sometimes necessary especially in complex fracture types to gain stable bone stock for press-fit cup implantation. Both implants, the suprapectineal plate and the cranially fixed revision cup, request high surgical experience and expertise and are mostly utilized in specialized trauma or orthopedic centers.

Our results demonstrate that the integrated flange in the revision cup reduced the interfragmentary movement to the same extent than the revision cup with additional suprapectineal plating. A trend toward increased stability was found in the CP group at the supraacetabular region, however not statistically significant. For both groups, gap movements in the supraacetabular region remained below 1 mm and in the acetabular region even below 0.5 mm. In the previous literature, a direct comparison of cementless cranial socket revision cups in combination to revision cups and plating is missing until now. In similar fracture patterns investigating only plate fixation, comparable studies revealed similar amounts of interfragmentary movements [17, 18].

In most of the tested specimens, the gap further opened, while in some specimens, the gap closed during loading. This can be explained by the fact that the fracture was cut manually with an oscillating saw, which resulted in a slightly uneven fracture surface. Thus, a perfect fracture reduction was hardly achieved and allowed also for gap closing in some specimens. In the supraacetabular region, higher gap movements were measured than in the acetabular region. Due to the chosen physiological load application of heel strike, the resulting load vector points predominantly toward the supraacetabular region, which led to highest movements in this area. For a different load scenario, the resulting load vector might slightly change and result in a different distribution of gap movement, but this has to be investigated in further biomechanical studies. Implant fixation of the revision cups was realized by three screws in the CP group and two screws and the additional fixation peg in the CF group. This guaranteed the same number of fixation options for both tested groups, as smaller sized revision cups only have three options for screw placement. Additionally, all screws were placed in the integrated flange. Screw arrangement in plate fixation was executed according to biomechanical expertise and clinical experience of the surgical team. Each plate was fixed the same way with three screws anteriorly und three posteriorly. Just the position of the additional infraacetabular screw needed to be adjusted in each specimen due to the individual anatomy of the pelves. In vivo this screw cannot always be drilled, as there is a risk of intraarticular placement in thin bone and a high surgical expertise is fundamental. The infraacetabular screw leads to compression of the fracture gap and to higher construct stability, which sometimes is difficult to achieve in vivo, where the screw placement is more demanding due to surrounding soft tissue.

Plate implantation led to better reduction results in the CP group, which could be seen in complete closing of the fracture gap at the ramus pubis inferior. An opening of the fracture gap during cup implantation was prevented by the plate and especially by the infraacetabular screw. In the CF group, the forces during cup implantation could not be totally neutralized by two k-wires and the fracture gap opened in some specimens. However, a minor opening of the gap during press-fit implantation represents a clinically realistic surgical procedure. In vivo initial press-fit is ensured by the surrounding soft tissue and by oversizing the revision cup in an intact iliac bone. In case of a comminuted supraacetabular region, press-fit is not the main task of the revision cup with integrated flange.

To further mimic a clinically relevant and physiological load pattern, our test setup combines the tilt of the pelvis at initial contact [12] with the respective force vector acting in the hip joint [13]. The inverted setup represents a realistic load scenario at heel strike with an applied load from the femur. The stepwise increasing load protocol started at partial weight-bearing loads and covered full as well as excessive weight-bearing up to failure.

The mode of failure was similar for both groups, and only in four specimens, the cup loosened. Due to this low number, no reasonable conclusion on implant failure can be drawn, but with three cases of cup loosening in the CP group and only one in the CF group, the integrated flange seems to be slightly advantageous in terms of implant stability. The region around the embedded sacro-iliac joint comprises the weakest point of the construct, as 7 out of 18 specimens failed due to breakage of the bone in this area. This failure mechanism can be attributed to the reduced bone mineral density of the used specimens and the limitation of other embedding options for this specific setup. In one CP sample, screw breakage right below the screw head occurred at the cup hole closest to the fracture line. This type of failure is also occasionally seen in clinical routine.

Biomechanical in vitro studies have the inherent limitation that in vivo situations including soft-tissue conditions and healing processes cannot be simulated. Although no muscle forces were considered in this setup, a clinically relevant and reliable load scenario was represented by heel strike [12, 13]. The load was physiologically applied via the femoral head into the acetabulum and was transmitted to the sacrum and the symphysis.

The idealized fracture line is both a limitation and strength of this study. In elderly patients, anterior column acetabular fractures often additionally extend into the anterior wall in combination with a compression and comminution of the weak bone [4]. The smooth fracture surfaces created with the oscillating saw might not totally represent a typical osteoporotic fracture in geriatric patients, but allow for a strictly reproducible approach and reasonable comparison of the implanted constructs. Another limitation is that in this idealized biomechanical setting, k-wires were used to substitute the stabilizing role of the surrounding soft tissue and guarantee proper handling. The k-wires might have been advantageous in the CF group, whereas in the CP group, further stability was provided by additional plate osteosynthesis. For mechanical testing, the k-wire through the spina iliaca anterior superior into the os ilium was not removed after implantation and further enhanced the stability. As long as this was consistent for all specimens, it did not affect the interpretation of results. For this study, we chose an anterior column fracture as it is one of the most common fracture types [3, 4], which can be primarily stabilized via THA. Fractures of the posterior, i.e., posterior hemi-transverse in combination with an anterior column fracture, are with nearly 20% the most frequently diagnosed fracture pattern in elderly patients [3]. This fracture type is rarely addressed with a primary THA. Our pre-study confirmed that this fracture type with anterior column and posterior hemi-transverse cannot be addressed in a biomechanical bone model without additional osteosynthesis, although it is reported in vivo [19]. A single cup construct cannot fix the posterior inferior fragment and has not enough osseous containment to provide an initial press-fit. Therefore, the primary THA is not suitable in a biomechanical model of anterior column in combination with a posterior hemi-transverse fracture. Based on the present results, we conclude that THA is an option in selected patients with a fracture limited to the anterior column and present osteoarthritis of the hip. These patients might benefit from early mobilization without secondary limitations due to posttraumatic exacerbation of the osteoarthritis, pain, and secondary implantation of a THA [20].

In conclusion, from a biomechanical point of view, both surgical approaches for simple anterior column fractures of the acetabulum showed comparable fracture reduction in terms of interfragmentary movement and construct stiffness. These findings support the option to treat acetabular fractures with concomitant osteoarthritis of the hip with an acute THA as presented in the current study. The potential advantages of less surgical trauma have to be demonstrated in clinical studies.

## Data Availability

Data are made available upon request.
